# Quantitative proteomic analytic approaches to identify metabolic changes in the medial prefrontal cortex of rats exposed to space radiation

**DOI:** 10.3389/fphys.2022.971282

**Published:** 2022-08-26

**Authors:** Evagelia C. Laiakis, Maisa Pinheiro, Tin Nguyen, Hung Nguyen, Afshin Beheshti, Sucharita M. Dutta, William K. Russell, Mark R. Emmett, Richard A. Britten

**Affiliations:** ^1^ Department of Oncology, Lombardi Comprehensive Cancer Center, Georgetown University, Washington, DC, United States; ^2^ Department of Biochemistry and Molecular & Cellular Biology, Georgetown University, Washington, DC, United States; ^3^ Division of Cancer Epidemiology and Genetics, National Cancer Institute, Rockville, MD, United States; ^4^ Department of Computer Science and Engineering, University of Nevada, Reno, NV, United States; ^5^ KBR, Space Biosciences Division, NASA Ames Research Center, Moffett Field, Mountain View, CA, United States; ^6^ Stanley Center for Psychiatric Research, Broad Institute of MIT and Harvard, Cambridge, MA, United States; ^7^ Department of Obstetrics and Gynecology, Eastern Virginia Medical School, Norfolk, VA, United States; ^8^ Department of Biochemistry and Molecular Biology, University of Texas Medical Branch, Galveston, TX, United States; ^9^ Department of Radiation Oncology, University of Texas Medical Branch, Galveston, TX, United States; ^10^ Department of Radiation Oncology, Eastern Virginia Medical School, Norfolk, VA, United States; ^11^ Department of Microbiology and Molecular Cell Biology, Eastern Virginia Medical School, Norfolk, VA, United States; ^12^ Center for Integrative Neuroinflammatory and Inflammatory Diseases, Eastern Virginia Medical School, Norfolk, VA, United States

**Keywords:** proteomics, space radiation, medial prefrontal cortex, quantitative, rats

## Abstract

NASA’s planned mission to Mars will result in astronauts being exposed to ∼350 mSv/yr of Galactic Cosmic Radiation (GCR). A growing body of data from ground-based experiments indicates that exposure to space radiation doses (approximating those that astronauts will be exposed to on a mission to Mars) impairs a variety of cognitive processes, including cognitive flexibility tasks. Some studies report that 33% of individuals may experience severe cognitive impairment. Translating the results from ground-based rodent studies into tangible risk estimates for astronauts is an enormous challenge, but it would be germane for NASA to use the vast body of data from the rodent studies to start developing appropriate countermeasures, in the expectation that some level of space radiation (SR) -induced cognitive impairment could occur in astronauts. While some targeted studies have reported radiation-induced changes in the neurotransmission properties and/or increased neuroinflammation within space radiation exposed brains, there remains little information that can be used to start the development of a mechanism-based countermeasure strategy. In this study, we have employed a robust label-free mass spectrometry (MS) -based untargeted quantitative proteomic profiling approach to characterize the composition of the medial prefrontal cortex (mPFC) proteome in rats that have been exposed to 15 cGy of 600 MeV/n^28^Si ions. A variety of analytical techniques were used to mine the generated expression data, which in such studies is typically hampered by low and variable sample size. We have identified several pathways and proteins whose expression alters as a result of space radiation exposure, including decreased mitochondrial function, and a further subset of proteins differs in rats that have a high level of cognitive performance after SR exposure in comparison with those that have low performance levels. While this study has provided further insight into how SR impacts upon neurophysiology, and what adaptive responses can be invoked to prevent the emergence of SR-induced cognitive impairment, the main objective of this paper is to outline strategies that can be used by others to analyze sub-optimal data sets and to identify new information.

## Introduction

The upcoming missions to Mars will present a number of challenges to the health of the astronauts. Due to inherent limitations of the spacecraft design and uplift capacity, space radiation (SR) exposure will be an unavoidable flight stressor on such missions. Using the current spacecraft design specifications, it is expected that astronauts will be exposed to ∼350 mSv/yr of SR during each year of the mission (Zeitlin et al., 2013; Iosim et al., 2019; Afshinnekoo et al., 2020)**.** Moreover, the current prediction of the “Local-Field” spectrum (the SR spectrum that the internal organs of astronauts will receive within the spacecraft) suggests that the majority of the physical and dose equivalent SR dose will arise from Z < 15 particles (Slaba et al., 2016; Simonsen et al., 2020).

Astronauts on deep space missions will have to act more autonomously than ever before due to the long lag time for communication between the space craft and Earth. For example, astronauts will have to solve critical unexpected problems by themselves to a much greater extent than on previous lunar or missions to the International Space Station (ISS). Creative problem solving utilizes several executive functions involved in planning, organization, decision making, judgment, task monitoring, attention, hypothesis generation, abstract thinking, and cognitive flexibility (Stuss and Levine, 2002; Cato et al., 2004; Sue Baron, 2004; Spinella, 2005). Regrettably, ground-based rodent experiments suggest that exposure to ≤25 cGy of several SR ions (i.e., protons, ^4^He, ^16^O, ^28^Si, ^48^Ti and ^56^Fe) impairs various aspects of executive function but primarily cognitive flexibility tasks (Britten et al., 2014; Davis et al., 2014; Parihar et al., 2015; Britten et al., 2018; Jewell et al., 2018; Parihar et al., 2018; Acharya et al., 2019; Britten et al., 2020a; Britten et al., 2020b; Whoolery et al., 2020; Britten et al., 2021a; Burket et al., 2021; Soler et al., 2021; Britten et al., 2022).

There is a comprehensive body of data on the effect that a wide spectrum of SR species has on performance in the attentional set shifting (ATSET) assay (Parihar et al., 2016; Britten et al., 2018; Britten et al., 2020a; Britten et al., 2021b; Burket et al., 2021). These data sets are now being analyzed with machine learning assisted computational approaches to fully characterize the cognitive deficits induced (Matar et al., 2021; Prelich et al., 2021). However, a readily identifiable consequence of SR exposure is the loss of performance in the Simple Discrimination (SD) stage of the ATSET test. Performance within the SD stage is primarily regulated by the mPFC (Bellone et al., 2015). The SD stage interrogates the rats’ decision making abilities, specifically associative recognition memory formation. This is an essential process in identifying (and learning) the salient (go/no-go) in a task. Should similar effects occur in humans, astronauts would experience a decreased ability to identify and maintain focus on relevant aspects of the task being conducted.

While at the cohort levels, SR exposed rats have a significantly worse ATSET performance than their unirradiated counterparts, there are marked inter-individual variations in the severity of ATSET impairments induced by SR (Jewell et al., 2018; Britten et al., 2020a; Britten et al., 2021a; Burket et al., 2021). Many of the SR-exposed rats had comparable performance to that seen in sham rats; but 30%–50% of SR-exposed rats have severely impaired performance metrics (less than the 5th percentile of sham cohort). These data suggest that some individuals are able to ameliorate the deleterious effects of SR while others are unable to do so. This bifurcating response of neurocognitive processes to SR exposure has important consequences for risk assessments, but also provides a unique opportunity to establish the impact of SR on neurophysiology, and the subsequent adaptive responses associated with the preservation or the impairment of neurocognition.

The mechanistic basis of SR-induced cognitive impairment remains largely unknown, but ultimately, such performance decrements are a reflection of the impact of SR exposure interfering with the ability of neurons to encode, store, retrieve, or actively extinguish memories. SR exposure does alter the functionality of neurons within multiple regions of the brain (Machida et al., 2010; Britten et al., 2014; Marty et al., 2014; Rudobeck et al., 2014; Bellone et al., 2015; Sokolova et al., 2015; Howe et al., 2019; Britten et al., 2020a; Krishnan et al., 2021), but emerging evidence suggests that these alterations may arise from the impact that SR has on both neuronal and non-neuronal cells. Astrocytes and oligodendrocytes play a critical role in regulating neuronal function through a variety of processes. For example, astrocytes play a critical role in regulating glucose metabolism and energy supply to neurons (Nortley and Attwell, 2017; Deitmer et al., 2019; Murphy-Royal et al., 2020), while oligodendrocytes are essential for providing metabolic support to neurons, rapidly transferring short-carbon-chain energy metabolites like pyruvate and lactate to neurons (Philips and Rothstein, 2017). The functionality of both of these cell types is impacted by SR exposure. Glutamate transporter activity in astrocytes is reduced after exposure to carbon and iron ions (Sanchez et al., 2010), while SR exposure leads to significant changes in the percentage of myelinated axons, suggesting that oligodendrocyte function is significantly impacted by SR exposure (Dickstein et al., 2018). In addition to these non-neuronal effects of SR exposure, at a systemic level there are elevated DNA methylation levels (reduced expression) in the hippocampus 1 month after SR exposure (Acharya et al., 2017), and SR also induces autophagy and persistent oxidative stress within the brain (Poulose et al., 2011), and widespread microglial activation (Krukowski et al., 2018b; Krukowski et al., 2018c; Raber et al., 2019; Ton et al., 2022).

Collectively, these studies indicate that SR exposure alters numerous processes within the brain. Taking all these factors into consideration, it seems likely that a systems biology approach will be necessary to identify why some individuals can still perform executive functions while others have impaired performance after SR exposure.

We have previously employed a robust label-free mass spectrometry (MS) based untargeted quantitative proteomic profiling approach to characterize the composition of the hippocampal proteome in juvenile (Britten et al., 2017) and adult (Dutta et al., 2018) male Wistar rats exposed to ≤20 cGy of 1 GeV/n^56^Fe. Nearly a quarter of the proteins found in the hippocampus of adult sham rats were lost or had reduced expression in the irradiated hippocampus (Britten et al., 2017). These data are consistent with the elevated DNA methylation levels observed in the hippocampus of rats exposed to 20 cGy ^28^Si ions at 1 month post exposure (Acharya et al., 2017). Approximately 10% of the proteins that were lost in the SR-irradiated rats are involved in various aspects of synaptic transmission including both pre- and post-synaptic proteins. These studies also identified proteins whose expression was altered in rats exposed to SR (radiation biomarkers), with a further subset of proteins whose expression was correlated with impaired spatial memory performance. These proteomic analyses clearly demonstrated that SR exposure impacted multiple aspects of the functionality of the hippocampus, and it appears that those rats that maintained a functional spatial memory after SR exposure lost fewer proteins than the rats that have impaired spatial memory, who also expressed proteins known to have a negative impact upon neuronal physiology.

It is unclear if SR-induced impairment of executive function performance (that is assessed by the ATSET test) is associated with similar proteomic changes as those observed in the hippocampus (Britten et al., 2017; Dutta et al., 2018). The marked inter-individual variation in the incidence and severity of ATSET impairment provides a unique opportunity to increase our understanding of how SR impacts upon neurophysiology and which pathways are altered when SR induces ATSET impairment, as well as identify the adaptive responses that prevent the emergence of ATSET impairment in some individuals. This study has established changes in the composition of the proteome from mPFC of adult male Wistar rats exposed to 15 cGy 600 MeV/n^28^Si ions and used four different approaches to mine the data to identify proteomic changes associated with impaired ATSET performance. As with many SR studies, there are severe logistical constraints that limit the availability of tissues for such analysis, and some of the strategies that can be applied to such limited data sets have been hindered due to low numbers of samples. Nonetheless, significant changes between sham and irradiated samples have identified perturbed proteins and pathways that can serve as basis for identification and development of countermeasures.

## Materials and methods

### Irradiation procedure

This study was conducted in accordance with the National Research Council’s “Guide for the Care and Use of Laboratory Animals (8th Edition),” at facilities of Eastern Virginia Medical School (EVMS) and Brookhaven National Laboratory (BNL), both of which are accredited by the Association for Assessment and Accreditation of Laboratory Animal Care, International. All procedures were approved by the Institutional Animal Care and Use Committee of both EVMS and BNL.

The rats used in this study are a subset of the 90 male Wistar retired breeder rats (HSD:WI; Harlan Sprague-Dawley, Inc., Indianapolis, IN, United States) that were used in our previous study (Britten et al., 2018). The rats were irradiated with 15 cGy 600 MeV/n ^28^Si exposure at the NASA Space Radiation Laboratory (NSRL) at BNL. Further details on acclimatization, transport, specific light cycles, and identification are described in detail in the previous study (Britten et al., 2018)**.**

### Attentional set shifting testing

At approximately 90 days post SR exposure the performance of the rats in the ATSET task was established according to our previously published protocol (Britten et al., 2018). The rodent ATSET task is a seven stage progressive test, where the rat has to form an association between the presence of the food reward and a physical cue (either the digging medium or scent). By altering the combination of scents and digging media, progressively more complex cognitive processes can be tested. The task requires sequential rule learning ability, utilizing information gained in a previous stage to solve the subsequent tasks. The rats were given a total of 30 trials to reach criterion (six consecutive accurate choices) at each stage. Any rat that did not reach criterion, or that scored an incomplete (did not make a choice within 3 min on three out of five consecutive trials) in any given stage, was assigned a Day 1 test score of 30 attempts to reach completion (ATRC), rested overnight and retested the following day. If the rat reached criterion on the second occasion, the aggregate ATRC score (30 for the first failure plus the number of attempts on the second day) was recorded and the rats were immediately tested in the next stage of the assay. If the rat failed to complete a stage on the second attempt, it was excluded from further analysis. Rats are sequentially tested for performance in the SD, Compound Discrimination (CD), Compound Discrimination Reversal (CDR), Intra-Dimensional Shifting (IDS), IDS Reversal (IDR), Extra-Dimensional Shifting (EDS) and EDS Reversal (EDR) stages of the test. All testing was conducted during the dark cycle while they were in their active stage, with the first rat being tested at ∼2 h into the 12 h dark cycle (Zeitgeber T+2). The time at which testing was commenced was kept constant for an individual rat. The ambient light within the testing room was only bright enough [4 Lux as determined by a Digital Lux Meter LX1330B (Kaysan Electronics, Mountain View, CA, United States)] for the observation of the rats.

### Medial prefrontal cortex protein extraction

**FIGURE 1:**
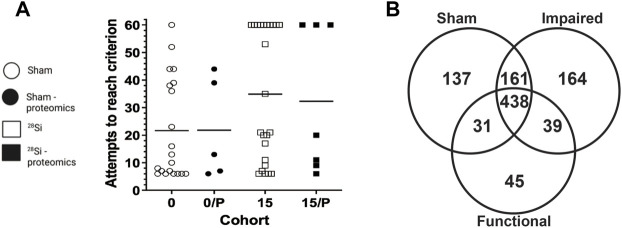
Effect of 600 MeV/n^28^Si -irradiation on performance of individual rats within the SD stage of the ATSET test **(A).** Individual attempts to reach completion criterion (ATRC) values for sham-irradiated rats (circles) or rats exposed to 15 cGy 600 MeV/n^28^Si (squares); horizontal bar denotes median ATRC value within a cohort. Closed symbols denotes rats that were used for the proteomic analysis. Cohort abbreviations: 0: all Sham-irradiated rats; 0/P: representative Sham-irradiated rats used for proteomic analysis; 15: all rats exposed to 15 cGy 600 MeV/n^28^Si; 15/P: rats exposed to 15 cGy 600 MeV/n^28^Si rats used for proteomic analysis. The Venn diagram **(B)** shows the number of proteins detected in the various groups.

After approximately a week from the completion of ATSET testing, rats were euthanized and the medial prefrontal cortex (mPFC) (along with several other brain regions) was recovered. Representative rats from each cohort [Sham *n* = 5, SR-ATSET high performers (Functional) *n* = 4 and SR-ATSET low performers (Impaired) *n* = 3] were selected for proteomic analysis based upon their SD performance status (Figure 1A). However, after completion of the proteomic analysis, it was decided that while two of the SR-ATSET high performer rats were proficient in the SD stage, given the fact that they failed to complete later stages in the ATSET test, they needed to be reclassified as SR-ATEST low performers, thus proteomic analysis was performed on the following cohorts [Sham *n* = 5, SR-ATSET high performers (Functional) *n* = 2 and SR-ATSET low performers (Impaired) *n* = 5].

To avoid inducing changes in the proteome of the mPFC due to anesthesia or asphyxiation, the rats were euthanized by guillotine. The brain was immediately recovered and the mPFC recovered in accordance with our previous protocol (Machida et al., 2010). The excised mPFC was placed in a sterile 1.5 ml Eppendorf tube, flash frozen in liquid nitrogen, and stored at −80°C until required for proteomic analysis. The protocol followed for peptide and protein identification for the brain tissue lysate has been published in a previous paper (Britten et al., 2017; Dutta et al., 2018). The mPFC samples were recovered from cryopreservation, weighed and placed in impact resistant tubes containing 6.5 mm garnet and ceramic sphere matrix (MP Biomedical, Santa Ana, CA, United States) with 1 ml of 8 M urea, 300 mM Tris-HCL, 10 mM DTT (pH 8.5) per 100 mg tissue sample. The sample was subjected to mechanical disruption in a FastPrep-24 instrument (MP Biomedical) for 20 s at a speed of 4m/s twice, the slurry was then centrifuged at 10,000xg for 10 min at 4°C and the supernatant transferred to a new microcentrifuge tube. The protein concentration of the supernatant was determined using a DTT compatible BCA assay (Thermo Fisher Scientific, San Jose, CA, United States) and 100 µg of extracted protein sample was run on a NuPAGE reducing gel (4%–12% Bis-Tris Gel) (Life Technologies, Carlsbad, CA, United States) with NuPAGE MOPS SDS 1X buffer run at 200 V for about 10 min. After the protein band had migrated 3–5 mm, the gel was stained with Page Blue (Bio-Rad, Hercules, CA, United States) and the entire protein band cut out. The gel was de-stained and washed three times in 50 mM NH4HCO3; 50% acetonitrile and 80% acetonitrile. The gel-bound proteins were reduced with 1 ml of 40 mM DTT for 25 min at 56°C. The gels were processed for LCMS analysis as described (Newton et al., 2012) and the recovered protein preparations shipped to University of Texas Medical Branch (UTMB) on dry ice. Upon arrival they were stored at 4°C prior to downstream analysis.

### Nanoflow liquid chromatography-tandem mass spectrometry analysis

Peptide mixtures were analyzed by nanoflow liquid chromatography-tandem mass spectrometry (nanoLC-MS/MS) using a nano-LC chromatography system (UltiMate 3000 RSLCnano, Dionex), coupled on-line to a Thermo Orbitrap Fusion mass spectrometer (Thermo Fisher Scientific, San Jose, CA, United States) through a nanospray ion source (Thermo Scientific) as described (Huang et al., 2020)**.** MS/MS spectra were extracted and charge state deconvoluted by Proteome Discoverer (Thermo Fisher, version 1.4.1.14). Deisotoping was not performed. All MS/MS spectra were searched against a Rat protein database (a total of 25,320 sequences) extracted from Swissprot (version 57) using taxonomy “Rattus”. Searches were performed with a parent ion tolerance of 5 ppm and a fragment ion tolerance of 0.60 Da. Trypsin was specified as the enzyme, allowing for two missed cleavages. Fixed modification of carbamidomethyl (C) and variable modifications of oxidation (M) and deamidation (N and E). Only those proteins that have >2 peptides identified (or >50% of protein covered by a single peptide) were included in the comparative quantitative analysis steps, and result in a correct protein identification probability of *p* < 0.05. A label-free precursor ion detection method (Proteome Discoverer, version 1.4, Thermo Scientific) was used because of the accurate mass measurements on proteins/peptides with specific retention times on precursors/fragments within 5 ppm mass accuracy. These factors combine to afford protein/peptide identifications with high confidence and high sequence coverage. The Sequest algorithm, a search engine employed by Proteome Discoverer (version 1.4, Thermo Scientific) was used to identify peptides from the resulting MS/MS spectra by searching against the combined Rat protein database (a total of 25,320 sequences) extracted from Swissprot (version 57) using taxonomy “Rattus”. Searching parameters for parent and fragment ion tolerances was set as 15 ppm and 80 mmu, trypsin was set as the protease with a maximum of two missed cleavages. Only those proteins that have >2 peptides identified (or >50% of protein covered by a single peptide) were included in the comparative quantitative analysis steps, and result in a correct protein identification probability of *p* < 0.05.

### Protein quantitation/triaging

Relative quantitation of a protein within a given technical replicate was achieved by calculating the area under the curve (AUC) for the respective de-isotoped peptide and charge reduced multiple tryptic peptides. A protein was classified as being “present” if it was identified in two of the three technical replicate samples for an individual rat mPFC sample. In the event that a protein was not detectable in a particular rat, an AUC value of one was assigned for that protein. The mean AUC value for each individual rat was then calculated. A mean cohort AUC value (and the SEM) was then calculated for any protein that was “present” in the majority of the individual rats within that cohort. In those instances where a protein was not detected in the majority of individual rats or when the SEM exceeded the mean AUC, those proteins were removed from further analysis. Proteins were classified as being up or downregulated compared to the sham-irradiated cohort levels by comparing the mean AUC for a protein from the rats within each irradiated cohort to the comparable data from the sham-irradiated cohort. The Wilcoxon-Mann-Whitney test was used to identify proteins whose expression differed from that seen in the sham-irradiated rats at the 5% significance level.

### Analysis A—MetaboAnalyst

Sample outliers and duplicate proteins were removed from the dataset prior to post-processing. Principal component analysis (PCA) was conducted with an in-house software in Python. Sham samples were compared to Impaired and Functional, together constituting the irradiated group. The percent percentage cutoff of presence in each group was set to 70% and Pareto scaling was implemented, in addition to linear correlation. Further analysis was conducted with the software MetaboAnalyst 5.0 (Chong et al., 2019; Pang et al., 2021). While this software has been used extensively in the field of metabolomics and lipidomics, the statistical and data analysis approaches can be adopted for analysis of proteomic data. Two analyses were conducted: Sham vs SR (F + I), and Sham vs. I, as F contained only two samples. Missing values were replaced by 1/5 of the minimum positive value of each variable**.** No data filtering or transformation were applied, and samples were normalized by the median. Pareto scaling was also applied. Fold change analysis was based on 1.5 cutoff and volcano plots implemented a 0.1 FDR corrected *p*-value. The volcano plot was constructed from the normalized and scaled data with the EnhancedVolcano package (Bioconductor) (http://bioconductor.org/packages/release/bioc/html/EnhancedVolcano.html). Heatmaps were created in R with pheatmap (https://github.com/raivokolde/pheatmap) through Euclidean distance, showing only the top 50 proteins based on the results from a *t*-test for Sham vs. Irradiated (F + I). These 50 proteins were further analyzed through a STRING network analysis to show protein-protein interactions. Graphical representation of identified proteins was conducted through the software GraphPad Prism 6. Gene Ontology Analysis was further conducted through PANTHER (Protein Analysis THrough Evolutionary Relationships) (Mi et al., 2010), based on the proteins with ≥1.5 fold change, biological and cellular component classification.

### Analysis B—mitochondrial specific analysis

MitoCarta 3.0 (Rath et al., 2021) was used to determine which protein expression data from the untargeted data was specifically mitochondrial related. Heatmaps were created in R with pheatmap (https://github.com/raivokolde/pheatmap) and lollipop plots were created in R with ggplot2 (H. Wickham. ggplot2: Elegant Graphics for Data Analysis. Springer-Verlag New York, 2016, see here: https://ggplot2.tidyverse.org/authors.html#citation). All proteins were included in the analysis.

### Analysis C—Consencus Pathway Analysis

Further data analysis and pathway enrichment was performed with the web-based platform Consencus Pathway Analysis (CPA) (Nguyen et al., 2021), modified for proteomic data. The k-nearest neighbor algorithm (*impute.knn*) (Hastie et al., 1999) was applied in this dataset in order to adjust for the missingness of the data, implemented in the *impute* R package to impute the missing values. Next, the data were rescaled using log2 transformation: $m=log_{2}\left(m+1\right)$ . The protein probes of the datasets were also mapped to Entrez IDs in order to perform enrichment pathway analysis. For a few proteins where multiple proteins are mapped to one Entrez ID (and vice versa), the average value was taken. The following comparisons were then performed: 1) *Functional* versus *Sham,* 2) *Impaired* versus *Sham,* and 3) *Functional* + *Impaired (both grouped as irradiated)* versus *Sham.* The Gene Set Enrichment Analysis (GSEA) software in R programming language (Mootha et al., 2003) was used to enrich gene sets downloaded from two databases: Kyoto Encyclopedia of Genes and Genomes (KEGG) (Kanehisa et al., 2016) and Gene Ontology (GO) (Ashburner et al., 2000; The Gene Ontology Consortium, 2019). The version 97.0 of *Rattus norvegicus* (rno) pathways were used for KEGG database, and the version 2021–01–01 of biological process namespace were used for GO database. Only gene sets with at least 15 genes were kept in the analysis. This resulted in 325 KEGG gene sets and 1,388 GO gene sets were included in the analysis. Each comparison using each database was run separately. This resulted in total six independent analyses. The statistical significance for dysregulated gene sets was determined by 1,000 permutations of the gene sets. Gene sets that have adjusted *p*-values (using FDR) smaller than 0.05 were considered as significantly impacted. A cross-comparison and meta-analysis were performed using an in-house web application (https://bioinformatics.cse.unr.edu/software/cpa/), which was visualized using CytoscapeJS (Franz et al., 2016).

### Analysis D—protein-protein interaction

Pathway enrichment analysis and visualization of the protein interactions were performed using a protein-protein interaction (PPI) network. For this part of analysis missing values were substituted with half of the lowest value within each group, while groups containing all missing values were substituted with the value 1. The analysis was restricted to proteins with fold-change >1.15 compared to Sham and performed enrichment pathway analyses for Impaired and Functional rats separately. The pathway enrichment analysis was done using PathDIP version 4.0.21.2 (Database version 4.0.7.0) (Rahmati et al., 2020). For this analysis we looked for enriched pathways among the rat-specific core pathways, from literature-curated databases, plus ortholog pathways, from protein orthologs annotated in human, plus extended pathways, were PathDIP integrates the previous two sets of pathways with direct PPI and predicts a species-specific network (extended pathways, with 0.99 confidence). Twenty-one pathway source databases were used, not including ACSN2 (Atlas of Cancer Signaling Network version 2) given its focus on cancer processes. Pathway enrichment *p*-values were adjusted using FDR and considered at a significance level of 0.05. For the PPI network visualization, all direct physical interactions were retrieved among proteins up- or down-regulated from Integrated Interactions Database (IID) (version 2018-11) (Kotlyar et al., 2019) and the PPI network was constructed with the software NAViGaTOR version 3.13 (Brown et al., 2009). Proteins were annotated in NAViGaTOR with Gene Ontology (GO) cellular localization.

### Data availability

All raw chromatographic data were uploaded to NASA’s GeneLab database (Ray et al., 2019) with accession number GLDS-505 DOI: 10.26030/9fzm-jc44.

## Results

The SD stage of the ATSET test assesses the decision making ability of the rats, i.e., their ability to form an attentional set on the correct associative cue (from a choice of two) for a food reward. The percentage of sham rats that passed the SD stage was 90.5%, but significantly less (60%) of the 15 cGy irradiated rats (*p* < 0.01, Chi-squared, two-tailed Fisher’s exact test) were able to complete the SD stage [30].

Figure 1A depicts the individual performance metrics (ATRC) for sham rats (circles) and rats exposed to 15 cGy 600 MeV/n 28Si (squares) (data reanalyzed from Britten et al., 2018). While the mean ATRC value for the Si-exposed rat cohort was significantly higher (*p* = 0.042, Mann-Whitney) than that of the sham cohort [Figure 1, (Britten et al., 2018)], some of the Si-exposed rats had performance metrics that fell below the median ATRC value for the sham cohort.

Representative rats from each cohort were chosen for proteomic analysis based upon their SD-ATRC metrics (Sham: *N* = 5, SR-ATSET high performers (*N* = 2) and SR-ATSET low performers: *N* = 5). After proteomic analysis was completed two SR-high performing rats were reclassified as low performing rats due to them failing to complete the CDR.

The composition of the mPFC proteome of the representative rats from each cohort (Sham-SR, 15/Impaired, 15/Functional) included proteins that reached our vigorous inclusion criteria (quantifiable in >66% of technical replicates, and present in >66% of the biological replicates) in the various cohorts of rats (Sham-functional: 767; 15/Functional: 554; 15/Impaired: 811 proteins). In some instances, a protein was not detected in a technical replicate, or in a biological replicate. A complete list of the identified proteins and names within each group is provided in the Supplementary Tables S1, S2.

Figure 1B depicts a Venn diagram of the proteins detected in the various cohorts. There were 438 proteins that were detected in all three cohorts, hereafter referred to as “common” proteins. It is possible to mine this data to identify proteins that are altered as a result of SR, or to identify proteins whose expression is associated with the rats’ ATSET (SD) performance ability. With regards to radiation specific changes in the mPFC proteome, there were 39 proteins that were detected in both the 15/Functional and 15/Impaired cohorts, but not the Shams, these proteins are hereafter referred to as “SR exposure” (SEM) proteins. The total number of proteins that showed ≥1.5 fold increase in the SR group compared to Sham were 404, while the total number of proteins that showed a ≤1.5 decrease in the SR group compared to Sham were 349. There were 137 proteins that were only detected in the Sham samples, i.e., they were not detected in either of the irradiated cohorts and 252 proteins that did not have a detectable level in the Sham group but were activated in the irradiated samples. The second aspect of our data mining was to identify proteins whose expression was associated with either impaired or functional ATSET performance. A notable feature of the SD performance data (Figure 1) is that ∼69% of rats retain apparently normal SD performance after SR exposure, which are denoted as 15/Functional rats. We identified 164 proteins that were only detected in the 15/Impaired rats and 45 proteins that were only detected in the 15/Functional rats. Within these analyses and overall data it is possible to identify key proteins that could explain the ATSET performance levels in the SR exposed rats. We further performed multivariate analyses using three distinct approaches.

### Analysis A

**FIGURE 2:**
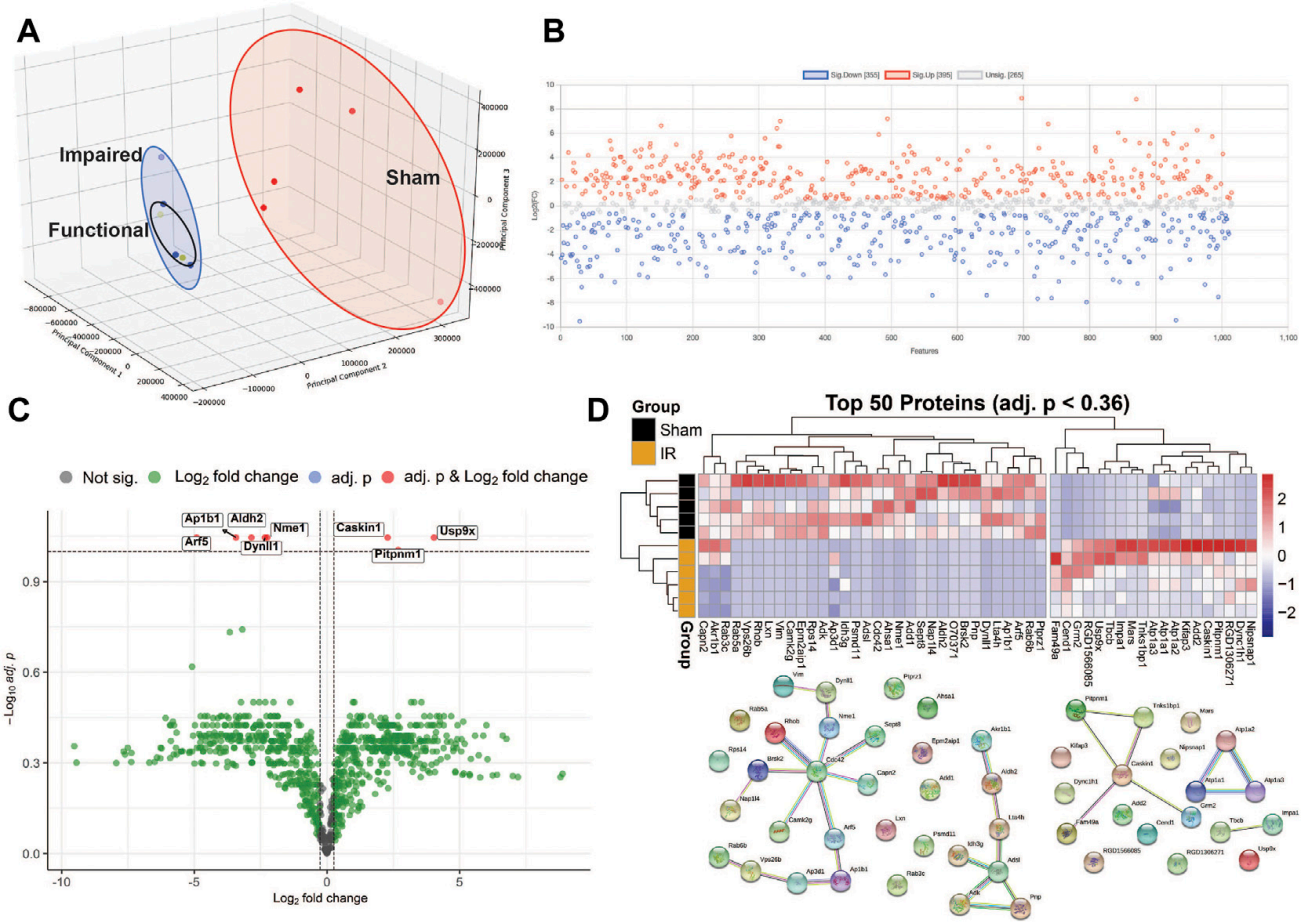
Multivariate data analysis. Panel **(A)** A 3D PCA scores plot shows that radiation is the main driver of the proteomic differences. Panel **(B)** Fold changes (1.5 cut-off) between exposed and sham. Panel **(C)** Volcano plot of exposed vs sham with fold-change of 1.5 cut-off and an FDR *p*-value of <0.1. Panel **(D)** Heatmap of the top 50 proteins and STRING network analysis of those proteins.

A PCA scores plot showed distinct clustering of sham and irradiated groups, with no discernible differences between functional and impaired groups (Figure 2A). This demonstrates that the primary overall separation is driven by exposure and that variability within a group decreases with radiation exposure. In addition, underlying protein expression levels that lead to behavioral differences are subtle in the overall protein content yet may be responsible for substantial outcomes in the exposed group (Figure 2A). Nonetheless, protein expression was perturbed as shown in Figure 2B with proteins with a fold changes of at least 1.5 (750 proteins out of 1,016). Of those proteins, only eight passed the criteria of high fold change and statistical significance (FC > 1.5, *p* < 0.05) (Figure 2C; Table 1), while a heatmap of the top 50 proteins with a *t*-test demonstrate the distinct expression levels between Sham and irradiated (Figure 2D). STRING network analysis of these top 50 proteins showed potential disruption of specific protein-protein interactions. Given the small n of the Functional group, multivariate analysis could not be performed on the three distinct groups. Nonetheless, the levels of the proteins from Table 1 showed two patterns: five proteins were completely ablated in the two irradiated groups, while three proteins showed a progressive increase with levels of dysfunction (Supplementary Figure S1). The ablated proteins are Nucleoside diphosphate kinase A, Aldehyde dehydrogenase (mitochondrial), AP-1 complex subunit beta-1, Dynein light chain 1 (cytoplasmic), and ADP-ribosylation factor 5. The three other proteins are Caskin-1, Ubiquitin specific peptidase 9 (X chromosome), and Membrane-associated phosphatidylinositol transfer protein 1.

**TABLE 1:** Proteins from volcano Plot.

Uniprot ID	Protein name	Fold change	log2(FC)	raw.pval	p.adjusted
P84083	ADP-ribosylation factor 5	0.033197	−4.9128	6.15E-04	0.089974
D3ZC84	Ubiquitin specific peptidase 9, X chromosome (Predicted)	16.401	4.0357	4.29E-04	0.089974
P52303	AP-1 complex subunit beta-1	0.09273	−3.4308	2.43E-04	0.089974
P11884	Aldehyde dehydrogenase, mitochondrial	0.13865	−2.8505	3.63E-04	0.089974
P63170	Dynein light chain 1, cytoplasmic	0.19775	−2.3383	6.10E-04	0.089974
D3ZE17	Caskin-1	4.8736	2.285	6.21E-04	0.089974
Q05982	Nucleoside diphosphate kinase A	0.2105	−2.2481	1.56E-04	0.089974
Q5U2N3	Membrane-associated phosphatidylinositol transfer protein 1	6.4259	2.6839	7.79E-04	0.098894

Based on the list of proteins with a 1.5 fold change, generated through MetaboAnalyst 5.0, functional classification analysis was performed through PANTHER (Supplementary Figure S2). Initial ontology on cellular components identified 14 categories of protein localization and functionality. Further investigation into cell parts identified roles in 21 categories and localizations with intracellular and membrane dynamics as the predominant areas. Interestingly, the oxidoreductase complex, and particularly the mitochondrial respiratory chain complex I and III showed perturbations in protein levels, that could lead to downstream perturbations in effective oxidative stress responses and energy production.

### Analysis B

**FIGURE 3:**
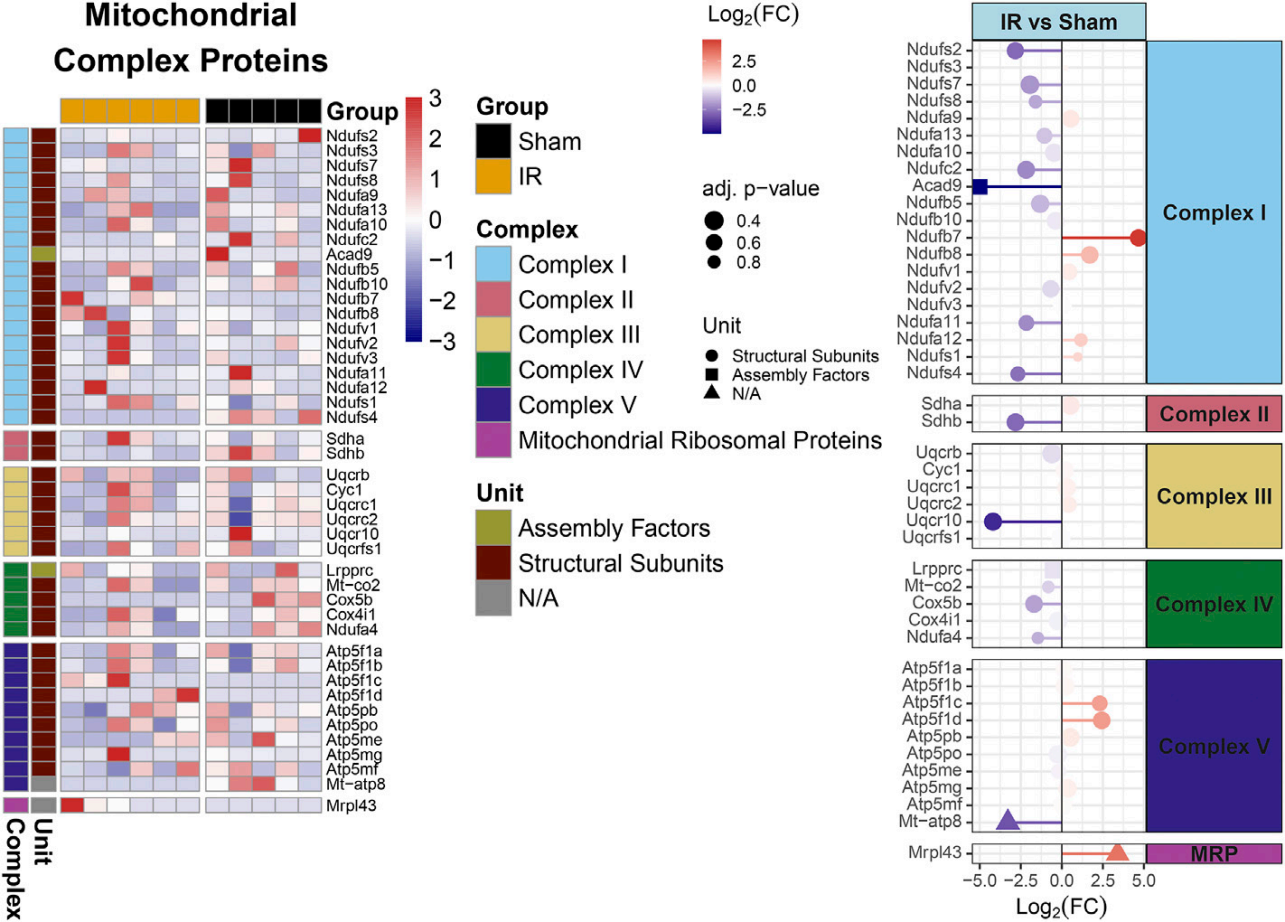
Mitochondrial OXPHOS complex proteins regulation comparing 15 cGy 600 MeV/n ^28^Si irradiated rats with sham. Heatmap of the protein expression for individual samples for each protein are shown on the left. Lollipop plots on the right, show the log_2_(Fold-Change) values with the adjusted *p*-values represented by the size of the size of the symbols and the shape of the symbols represent whether the proteins are the structural subunits (•), Assembly factors (■), or neither (▲). All complexes that are present with the data are shown.

**FIGURE 4:**
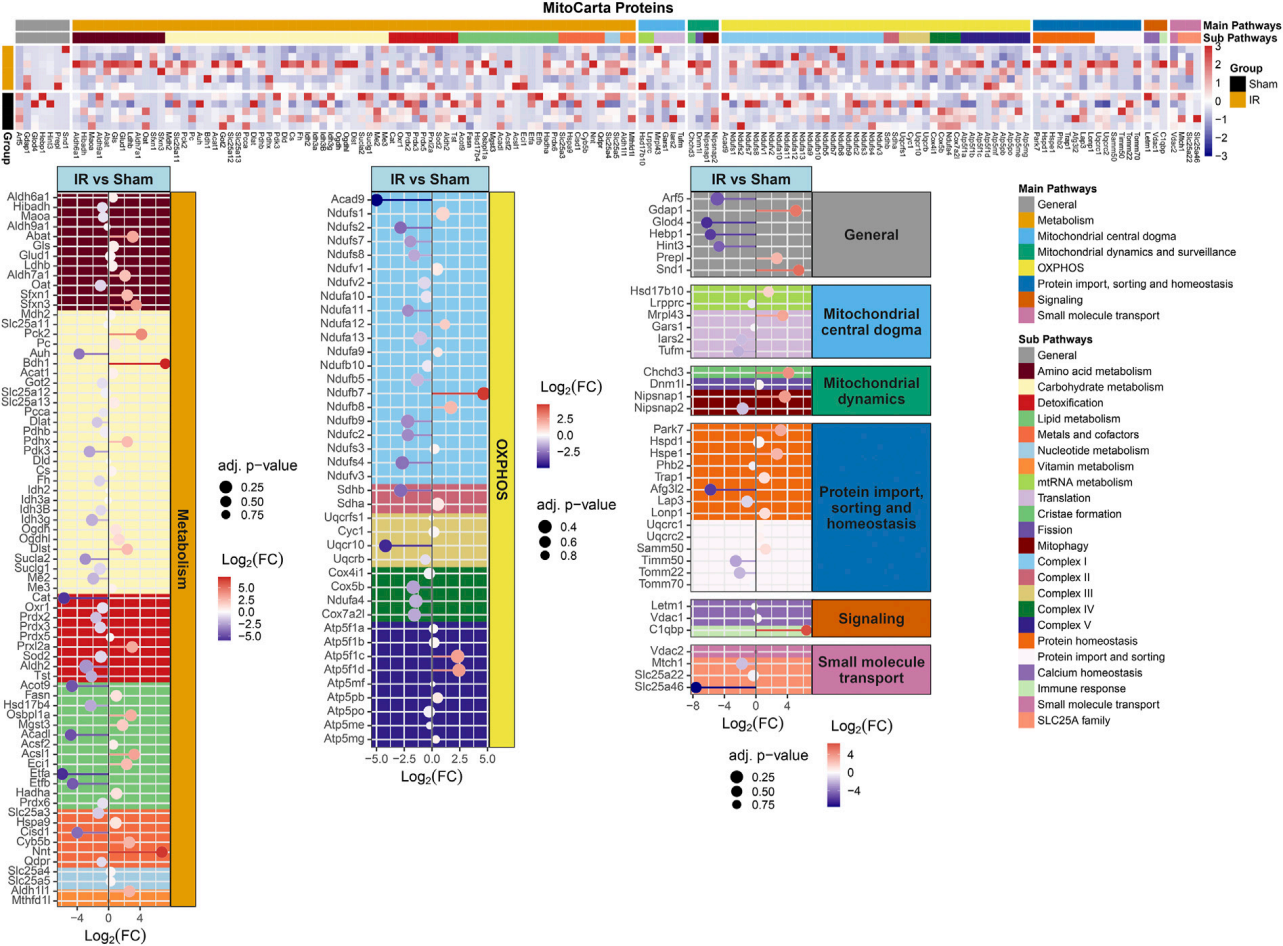
MitoCarta 3.0 genes overlapped with the proteins present for comparing 15 cGy 600 MeV/n^28^Si irradiated rats with sham. Heatmap of the protein expression for the MitoCarta 3.0 genes that are present for individual samples (top plot). The Main Pathway color bar represents the general MitoPathway categories for each protein. The Sub Pathway color bars show the detailed sub-categories for each pathway. Lollipop plots (bottom plots) show the log_2_(Fold-Change) values with the adjusted *p*-values represented by the size of the symbols for each of the proteins. The side facet represents the main pathway groups, while the background is colored to represent the Sub Pathways. Same color scheme is utilized for the lollipop plots as the heatmaps.

Protein levels from irradiated rats compared to sham showed an overall downregulation of oxidative phosphorylation (OXPHOS) complex proteins (Figure 3). Interestingly, complexes I and IV related proteins where the most represented. In addition, we observed the majority of the proteins related to mitochondrial metabolism (Figure 4) were also downregulated. Specifically, carbohydrate metabolism, lipid metabolism, and detoxification were the most suppressed in samples from irradiated animals.

### Analysis C

The results are presented by graphs in which nodes represent protein sets and edges represent the number of common proteins of two protein sets. Enrichment results in each comparison is encoded by a corresponding part in the pie chart inside each node, which represents a gene set. A colored part indicates that the pathway is significantly impacted in the corresponding analysis. The overall dataset contained 33% missing values, which were handled as described in the materials and methods.

**FIGURE 5:**
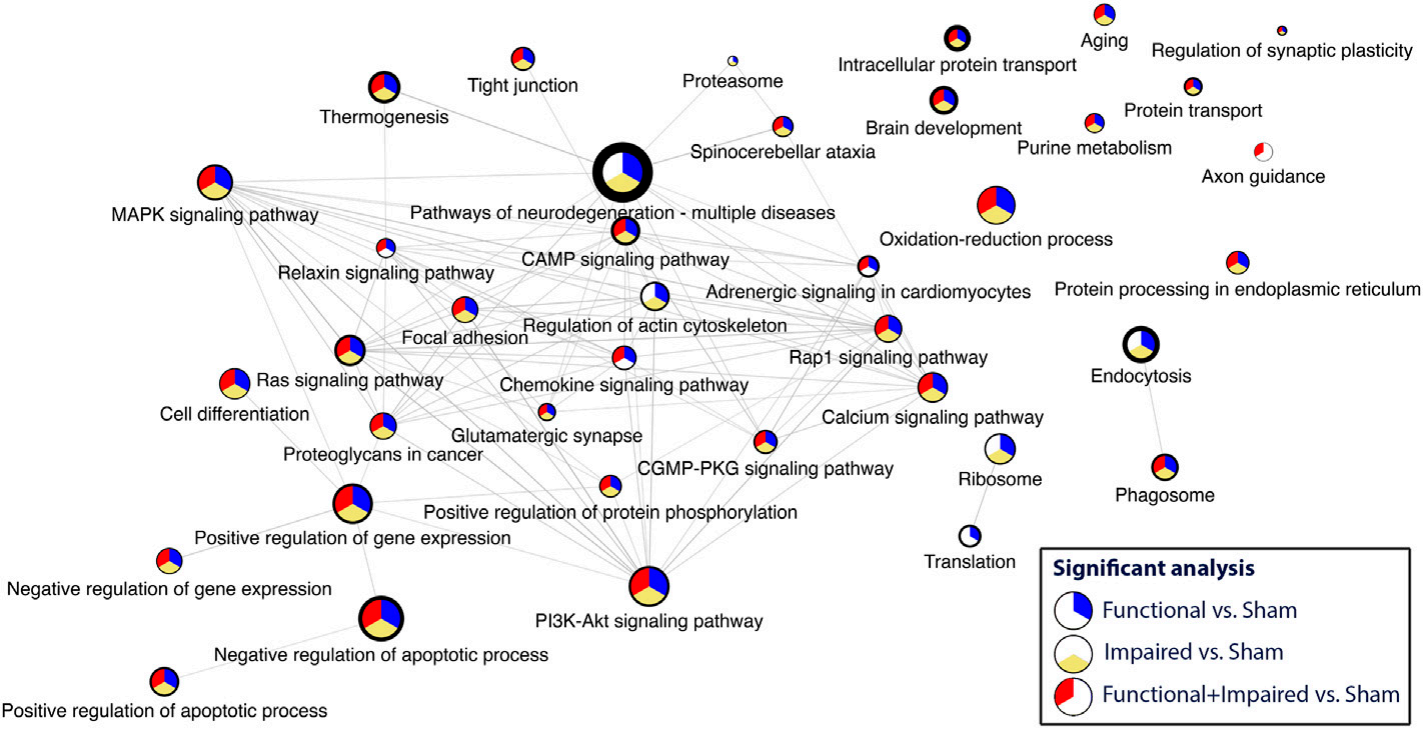
Consensus pathway analysis and visualization of enriched pathways with a GSEA<0.5 and a minimum of four statistically significant proteins per pathway. Higher enrichment is depicted through the border thickness. The colors blue, yellow, and red represent the significance of the three analyses: i) Functional versus Sham, ii) Impaired versus Sham, and iii) Functional + Impaired (both grouped as irradiated) versus Sham, respectively.

Criteria for inclusion included a GSEA of <0.05 and a minimum of four statistically significant proteins. Disease related pathways (e.g., Huntington, Alzheimer’s, Parkinson, viral response) were excluded from the network. The most enriched pathway was pathways of neurodegeneration. Thirty three pathways were included in the network (Figure 5, Supplementary Figure S3). Most enriched pathways identified through the degree of border thickness, included endocytosis, brain development, intracellular protein transport, purine metabolism, thermogenesis, and negative regulation of apoptosis. Select pathways (purine metabolism, axon guidance, focal adhesion, glutamatergic synapse, tight junction, and endocytosis) were further mapped along the KEGG pathways (Supplementary Figures S3–S7). One KEGG pathway, pathways of neurodegeneration (Supplementary Figure S8) showed perturbations along multiple different pathways, including the MAPK pathway, oxidative phosphorylation, Wnt signaling, and autophagy.

### Analysis D

**FIGURE 6:**
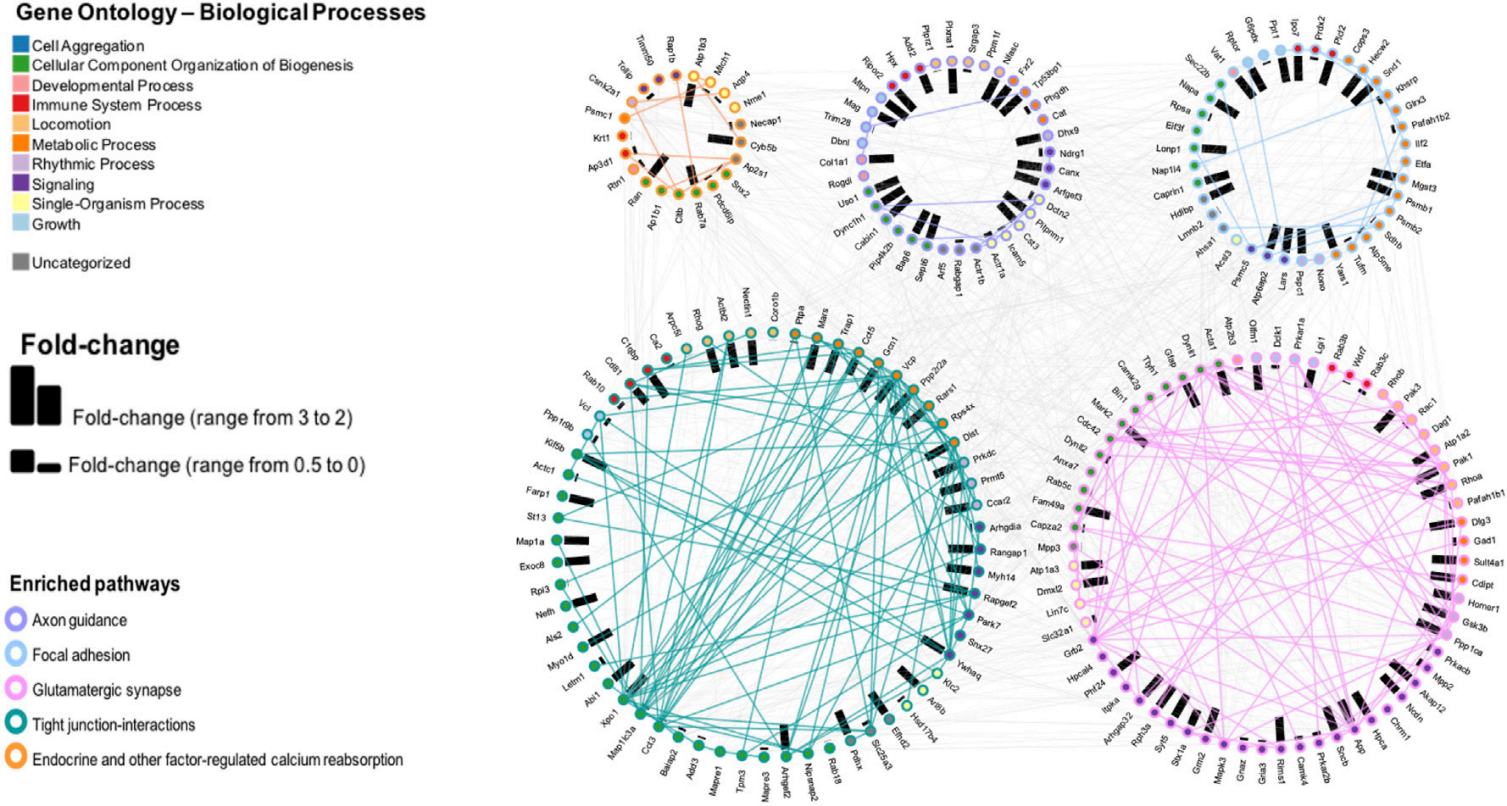
Pathway enrichment analysis and visualization of the protein interactions with a protein-protein interaction (PPI) network of five pathways selected from the CPA analysis. Fold changes are depicted by bars, representing change in either direction.

We depicted proteins with highest fold change (i.e., ≥2 in either direction). Analysis was concentrated on five pathways, as selected from Analysis C having the lowest *p* value. The results are shown in Figure 6. Samples from irradiated rats showed 103 proteins upregulated, while 324 proteins showed decreased levels. Further separation into functional or impaired compared to sham further highlighted the underlying differences present based on behavioral outcome. While impaired showed a higher number of increased proteins (220) vs. decreased proteins (19), the functional group had 50 increased vs 86 decreased total proteins. In this pathway enrichment analyses we have also identified the five pathways with the lowest *p* value identified in the previous analysis (Analysis B), therefore we selected these for visualization, including: axon guidance, focal adhesion, glutamatergic synapse, tight-junction interactions, and endocrine and other factor regulated calcium reabsorption. Within each pathway, fold changes of sham vs. SR are represented as bar graphs with weighted effect, and connecting lines represent protein-protein interactions. Importantly, based on gene ontology, each protein is also mapped to a biological process. Proteins were colored according to their gene ontology biological processes including cell aggregation, cellular component organization of biogenesis, developmental process, immune system, metabolic processes, rhythmic processes, signaling, single-organism processes, and growth, while a minority was uncharacterized based on this particular analysis and availability of data in the databases. Interestingly, the majority of proteins in the tight junction-interaction pathway were classified as cellular component organization and biogenesis, and the majority of proteins in the glutamatergic synapse pathway were classified as signaling. The top five proteins with highest number of PPI interaction in this network analysis were P62260 (Ywhae), P08592 (App), P62994 (Grb2), Q80U96 (Xpo1) and P35213 (Ywhab). Overall, SR had a significant effect in the protein levels of key intermediates in these pathways, that may influence normal function of the mPFC.

## Discussion

Future planned long duration missions to the Moon and Mars will inevitably expose astronauts to relatively high cumulative doses of high energy particles, as leaving low Earth orbit will eliminate some of the protection from the magnetosphere. These particles, due to their nature, have a higher biological relative effectiveness, with the potential to lead to significant adverse effects and higher risks for cancer, cardiovascular disease, and neurocognitive impairment, among others. In terms of cognitive effects, significant research efforts have identified and reproduced cognitive and behavioral decline in animal models (reviewed in (Cekanaviciute et al., 2018; Cucinotta and Cacao, 2019; Kiffer et al., 2019; Whoolery et al., 2020; Britten et al., 2021b)), and showing that SR leads to structural and molecular changes in the brain that can lead to altered behavioral patterns. Much of the work has focused on changes in the hippocampus in rodents, a brain structure with significant roles in memory and learning. In this study, we focused on the mPFC of the brain from rats exposed to an acute dose of 15 cGy of 600 MeV ^28^Si and assessed behaviorally at 90 days after exposure with the ATSET test. The mPFC’s were subsequently subjected to untargeted proteomic analysis to identify altered pathways from radiation exposure that could contribute to behavioral changes and potentially be targeted for development of appropriate countermeasures.

The mPFC plays a role in decision making, short and long-term memory and consolidation of time scales, attention, inhibitory control, habit formation and working (Jobson et al., 2021). Any disturbances therefore in the delicate interconnected molecular pathways may lead to significant effects in the structure itself and in other brain regions that are linked to mPFC, such as thalamus, amygdala, and hippocampus (Jobson et al., 2021). For example, the loss/downregulation of Drebrin-like protein or Syntaxin-7 in the SR-exposed rats could reflect SR-induced changes in dendritic architecture/synaptic plasticity (Takahashi et al., 2003; Mori et al., 2021). Similarly, the selective increase in the expression of GFAP in the 15/low performers could indicate that such rats have low performance due to elevated levels of gliosis (Yang and Wang, 2015; Ton et al., 2022). Ascribing biological significance to selectively sampled proteins is convenient but is fundamentally not scientific, relying upon the subjective bias of the investigator in the context of the experiment being performed. Furthermore, it must be remembered that multiple proteins within a process may need to be upregulated to alter the final “output” of that process, however, reduced expression of a single constituent protein within a pathway can often have a big impact on the final biological output of that pathway.

Proteomic analysis provides an untargeted evaluation of protein changes and network dysfunction that could impair normal cognitive processes. While proteomic data collection has been standardized in the last few years, data analysis still can employ different and unique methods of visualization and information extraction that can be borrowed from other -omics fields (e.g., transcriptomics, metabolomics). This offers the ability to build new tools that can incorporate results from various -omics analyses, such as the commercially available Ingenuity Pathway Analysis (QIAGEN), or software such as CPA that is utilized in our study (Nguyen et al., 2021). In this study we focused on proteomics of a select population of exposed rats in order to determine if any biological perturbations are a result of radiation exposure and provide a connection to the behavioral changes. Prior studies in hippocampus samples from rats exposed to 15 cGy of 1 GeV/n^48^Ti (Tidmore et al., 2021) identified a switch towards increased pro-ubiquitinated proteins in exposed animals.

Similarly to the observations in the hippocampus by Tidmore et al. (Tidmore et al., 2021), there was a significant number of proteins that showed depletion in the irradiated samples (Figure 1; Supplementary Figure S1) irrespective of behavioral outcome, while some proteins (Caskin-1, ubiquitin specific peptidase 9, and membrane associated phosphatidylinositol transfer protein 1 as examples, shown in Supplementary Figure S1) showed progressively increased levels, dependent on both irradiation and behavioral outcome. The Impaired group showed higher variability but overall higher levels than the other two groups. This indicates that there could be variable levels of dysregulation in a population that could be mitigated appropriately at early time points to maintain proper brain function. SR exposure however, with the specific conditions in this study, was the primary driving force in the overall proteomic changes and outcome stratification did not reveal global differences, as seen in a PCA scores plot (Figure 2A).

Applications of new methods of analysis, through CPA (Nguyen et al., 2021) and pathway enrichment and PPI, revealed critical pathways with high degrees of perturbations and enrichment. Pathways of neurodegeneration, brain development, and endocytosis (Figure 5) indicate that recycling of membranes after neurotransmitter release (Parton and Dotti, 1993) and decline in mechanisms of neuro-homeostasis could be a contributing factor to behavioral changes and should be further evaluated with additional -omics techniques to account for a collective profile of radiation exposure. Furthermore, impairment in proteins in neurotransmitter related pathways, such as glutamatergic synapse, calcium signaling pathway, and purine metabolism (Supplementary Figures S3, S5, S7) can have direct effects in behavior.

Furthermore, identifying the PPI within perturbed pathways, can lead to direct biological processes and hub proteins with high protein-protein interaction degrees that be targeted for countermeasure development. In this specific study, gene ontology analysis specific for biological processes revealed ten different processes that are affected by space radiation in mPFC. Two examples, immune system and metabolic processes can be explored for intervention (Krukowski et al., 2018a; Krukowski et al., 2021; Raber et al., 2021). Metabolic processes can also be linked to defects in mitochondrial respiratory chain and therefore overall mitochondrial dysfunction (Figures 3, 4, Supplementary Figure S2), which have been documented as a consequence of space radiation exposure and spaceflight (Barnette et al., 2021; da Silveira et al., 2020; Gan et al., 2018; Laiakis et al., 2021; Rubinstein et al., 2021), with persistent oxidative stress as a potential mechanism of contribution to brain dysfunction. In this study, carbohydrate metabolism, lipid metabolism, and detoxification were the most suppressed in samples from irradiated animals. The correct balance for the OXPHOS complexes in the mPFC is essential for maintaining the bioenergetics needed to prevent cognitive issues. Oxidative stress is essential for mitochondrial associated diseases (Wallace, 2013). Similar decreases with the OXPHOS complexes have been observed with aging and CNS related diseases (Bergman and Ben-Shachar, 2016; Park and Hayakawa, 2021; Takihara et al., 2015; van den Ameele and Brand, 2019). Interestingly it has been reported that decreases in OXPHOX complexes in neuronal cells lead to decreased proliferation and even impact neuronal stem cell functions (van den Ameele and Brand, 2019). Taken together, further studies in this area should include a comprehensive multi-omics analysis to specifically identify the level of long term changes to space radiation that will include small molecule quantification to measure neurotransmitter changes and link to behavioral effects.

While our study clearly has limitations due to the small number, it has provided unique methods of proteomic data analysis and identified pathways that could be further explored for countermeasure development. In addition, it only utilized a single acute dose and a single beam, which is not a true representation of the space radiation environment. Furthermore, radiation in addition to other stressors (e.g., microgravity, sleep deprivation, increased CO_2_ levels) may exacerbate the effects and therefore the altered behavioral patterns. Future studies should expand on multi-omic analyses as an initial step in developing a comprehensive view of the molecular changes that can lead to altered behavioral patterns that can significantly impact a long term space mission. The identified list of proteins and biological pathways from the mPFC is the first database of low dose space specific radiation. In combination with previous publications by our group on hippocampal proteins affected by low dose radiation, this publication adds to NASA’s GeneLab open science database of specific peptides that show dysregulation from different areas of the brain directly related to space relevant dose effects.

## Data Availability

The original contributions presented in the study are publicly available. This data can be found here: NASA GeneLab GLDS-505 (https://doi.org/10.26030/9fzm-jc44).

## Supplementary material

The Supplementary Material for this article can be found online at: https://www.frontiersin.org/articles/10.3389/fphys.2022.971282/full#supplementary-material

Supplementary Figure S1 Proteins identified through stringent criteria from the volcano plot of exposed vs. sham. The proteins were graphed based on functional and impaired categorization in the exposed group.supplementary-material.

Supplementary Figure S2 Gene Ontology Analysis through PANTHER based on cellular component classification and cellular localization.supplementary-material.

Supplementary Figure S3 KEGG pathway of the purine metabolism with identified protein perturbations.supplementary-material.

Supplementary Figure S4 KEGG pathway of the focal adhesion pathway with identified protein perturbations.supplementary-material.

Supplementary Figure S5 KEGG pathway of the glutamatergic synapse pathway with identified protein perturbations.supplementary-material.

Supplementary Figure S6 KEGG pathway of the tight junction pathway with identified protein perturbations.supplementary-material.

Supplementary Figure S7 KEGG pathway of the calcium signaling pathway with identified protein perturbations.supplementary-material.
