# Supplementary figures and images for: Quantitative proteomic analytic approaches to identify metabolic changes in the medial prefrontal cortex of rats exposed to space radiation
Source: Front Physiol. 2022 Aug 26;13:971282. doi: 10.3389/fphys.2022.971282 (PMC9459391; doi:10.3389/fphys.2022.971282)

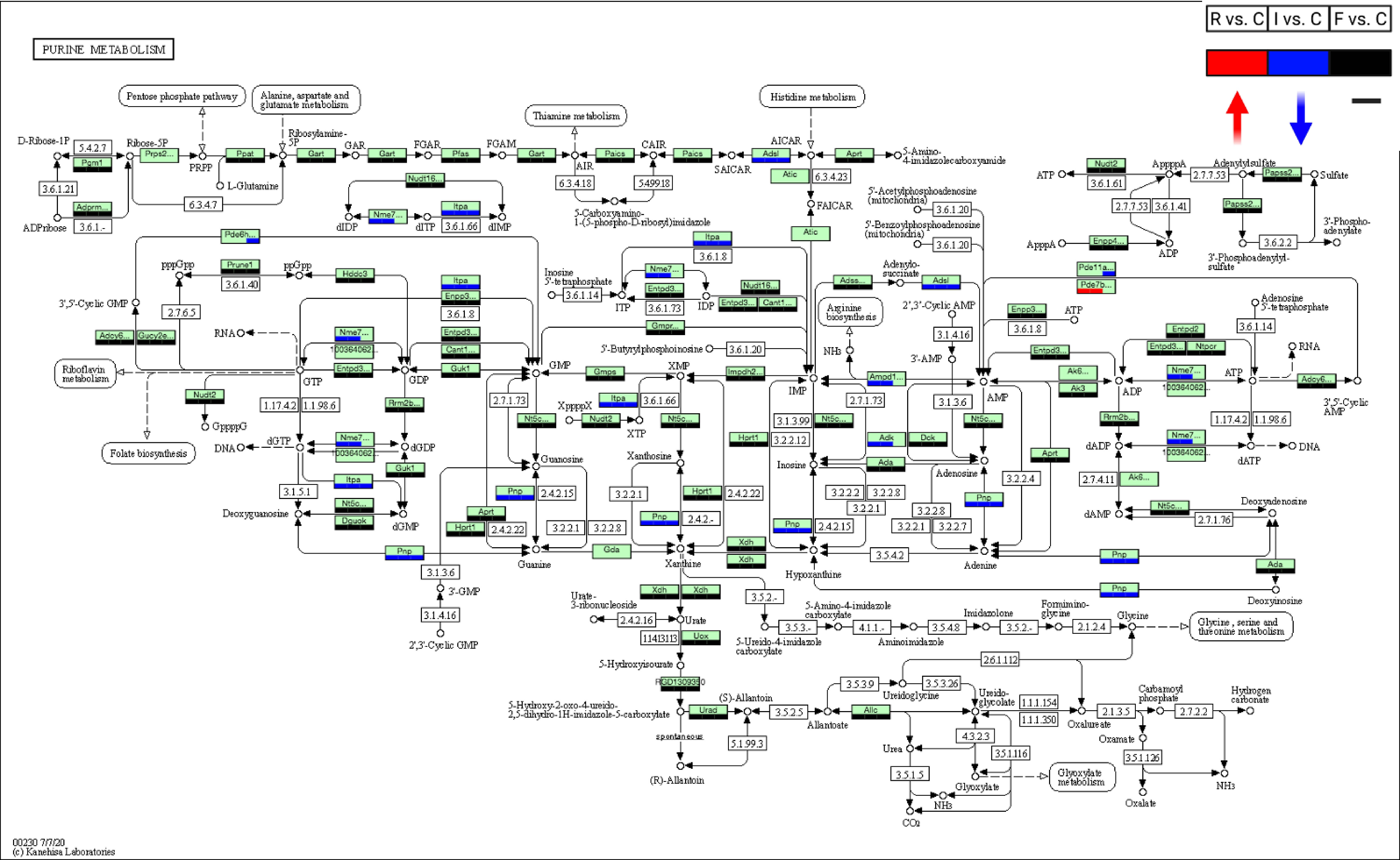

Supplement: Supplementary file 2 [file Image3.JPEG]

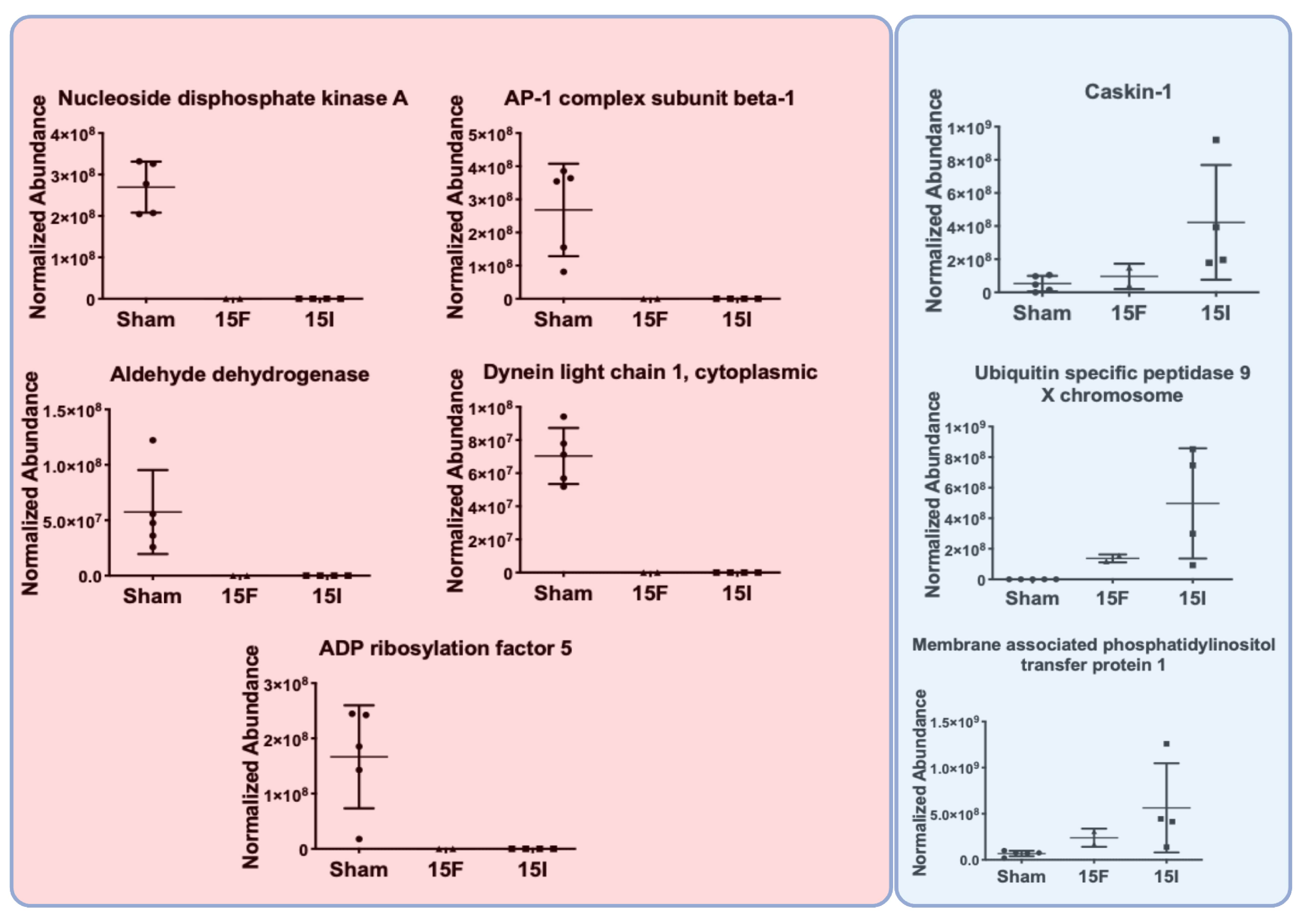

Supplement: Supplementary file 4 [file Image1.JPEG]

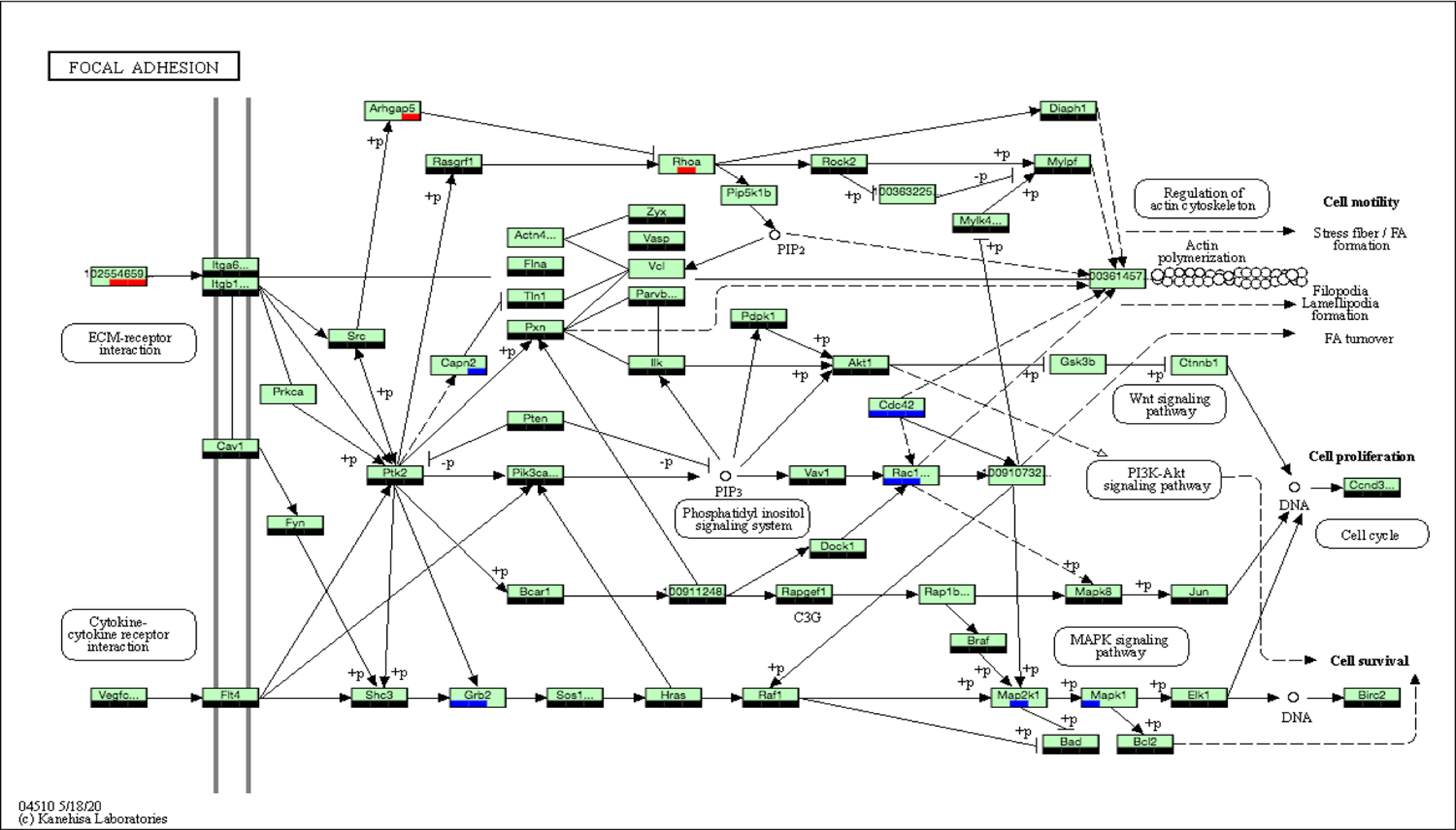

Supplement: Supplementary file 5 [file Image4.JPEG]

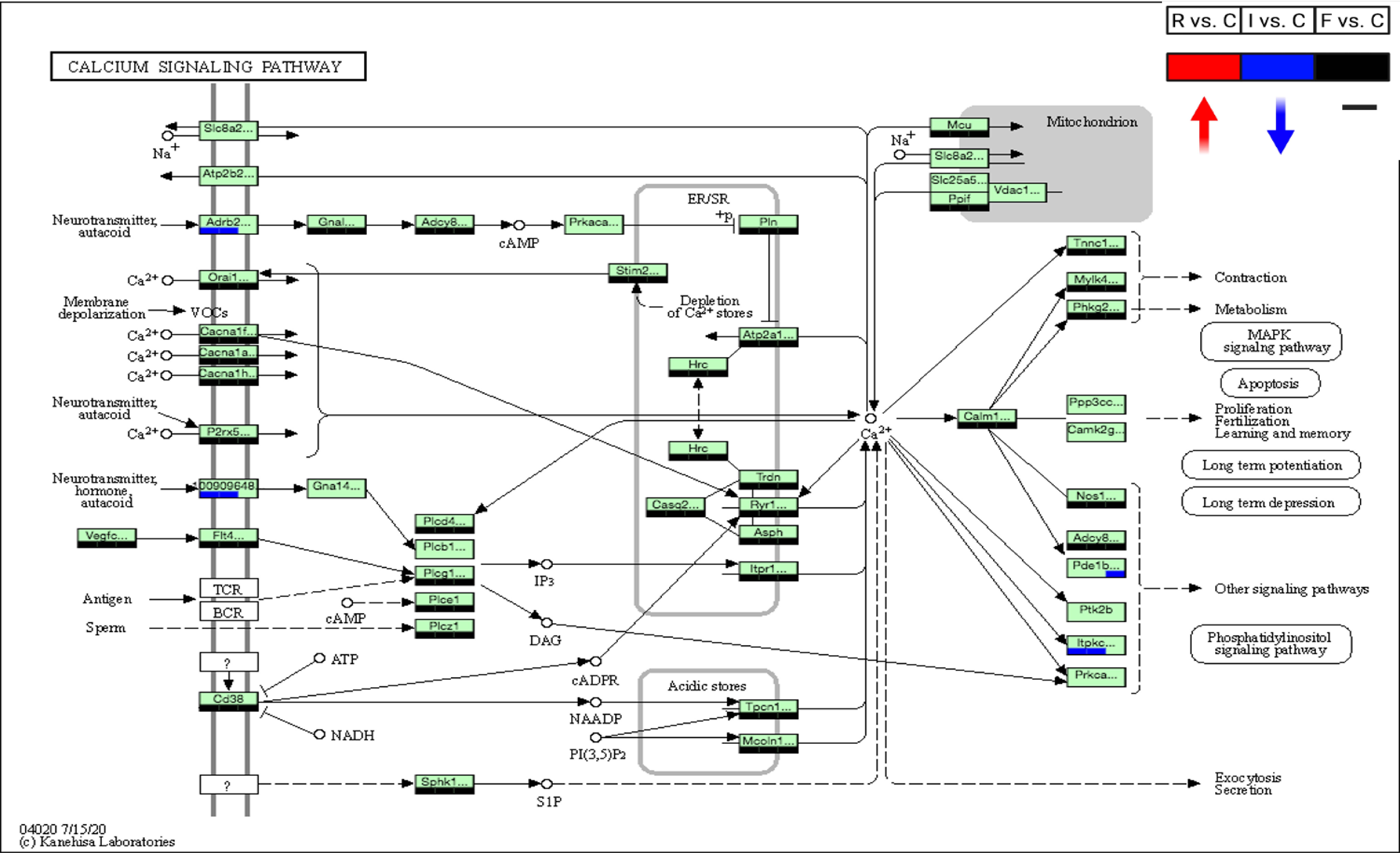

Supplement: Supplementary file 6 [file Image7.JPEG]

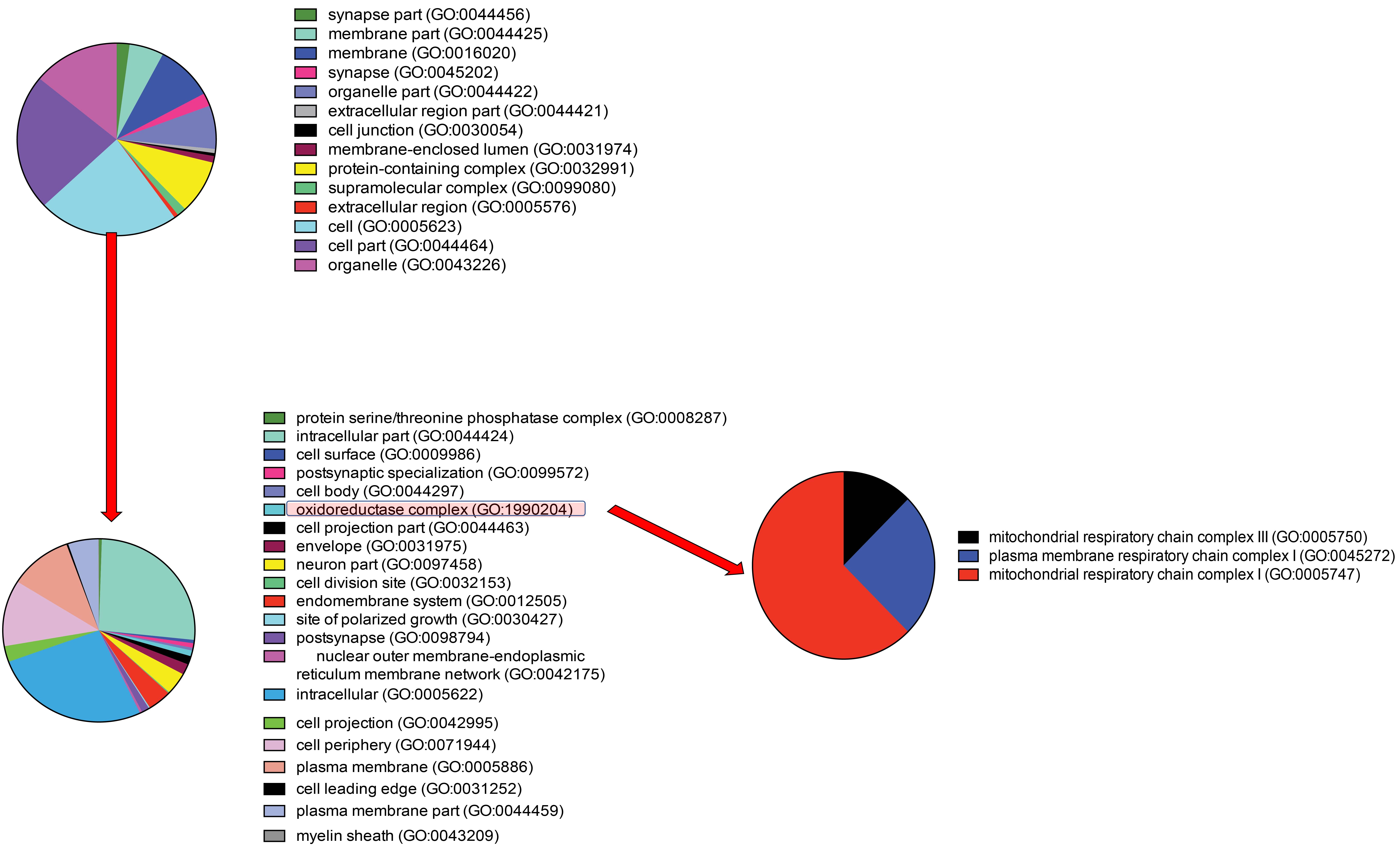

Supplement: Supplementary file 7 [file Image2.JPEG]

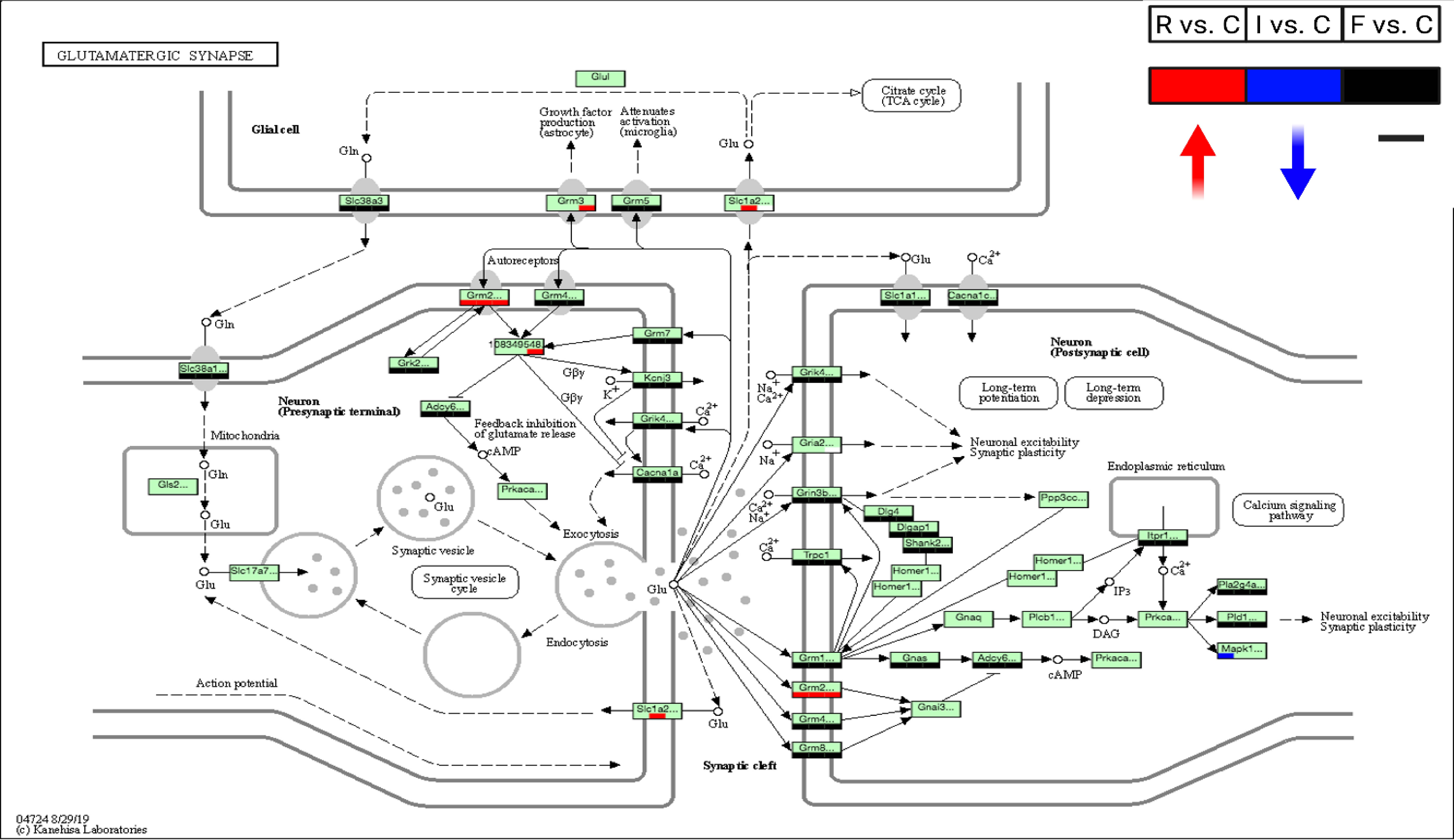

Supplement: Supplementary file 8 [file Image5.JPEG]

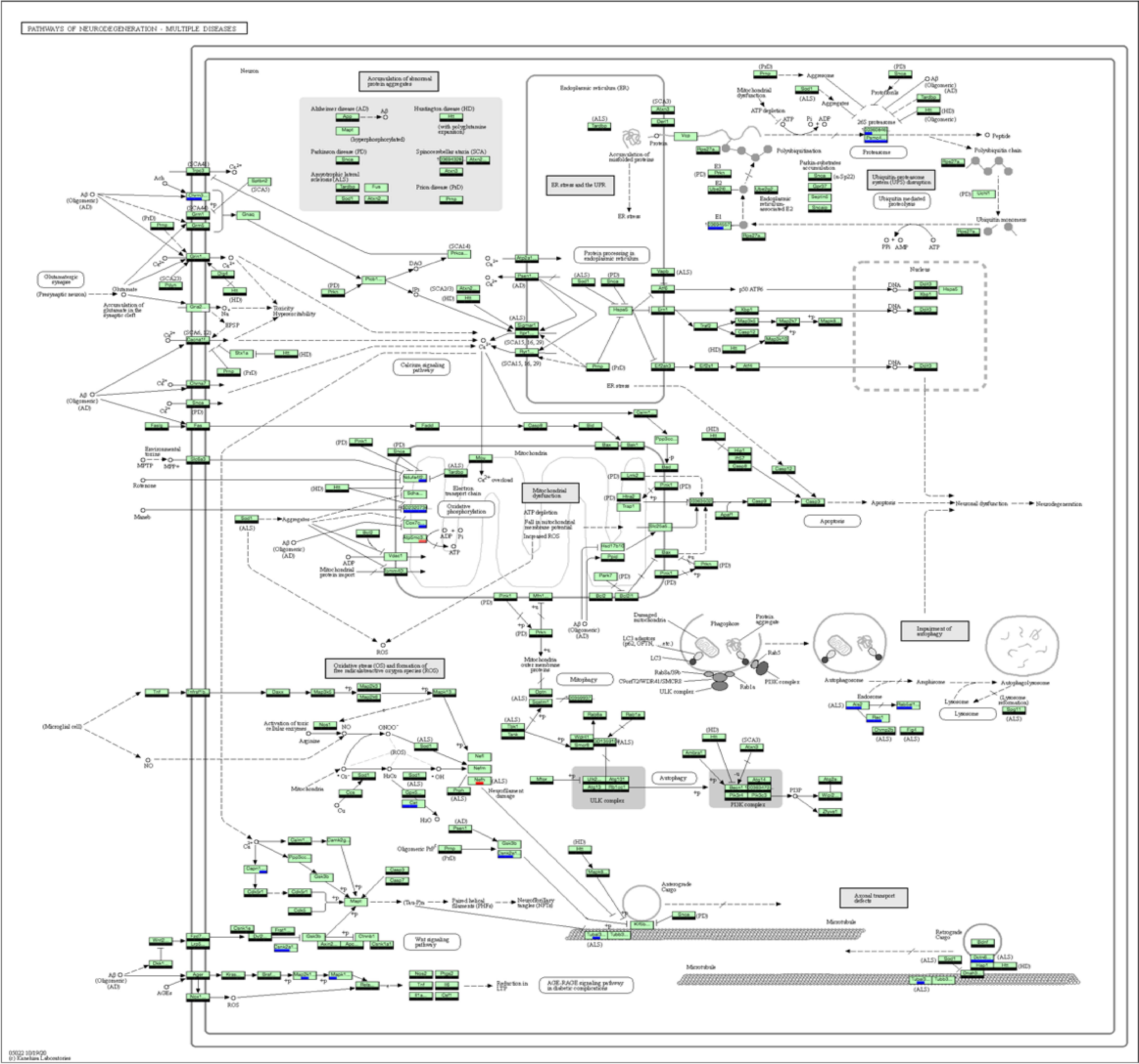

Supplement: Supplementary file 10 [file Image8.JPEG]

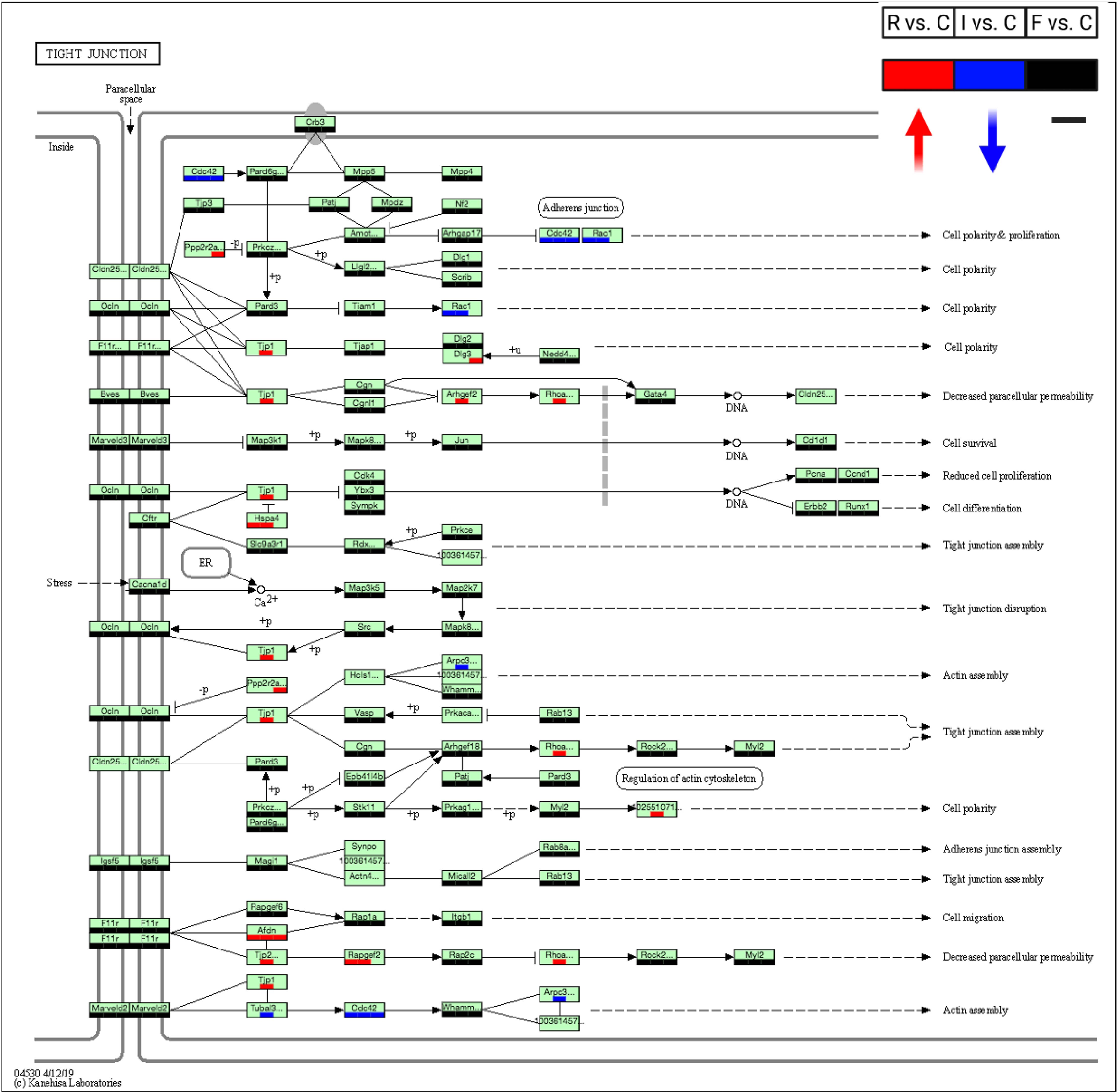

Supplement: Supplementary file 12 [file Image6.JPEG]
